# Quality of life and surgical outcome of ABBA versus EndoCATS endoscopic thyroid surgery: a single center experience

**DOI:** 10.1007/s00464-021-08361-w

**Published:** 2021-03-08

**Authors:** Ulrich Wirth, Thomas von Ahnen, Josef Hampel, Josefine Schardey, Peter Busch, Hans Martin Schardey, Stefan Schopf

**Affiliations:** 1 Department of General, Visceral, and Transplant Surgery, Ludwig-Maximilians-University Munich, Marchioninistr. 15, 81377 Munich, Germany; 2 Department of General, Visceral, Vascular and Endocrine Surgery, Agatharied Hospital, Hausham, Germany; 3 Department of General Surgery, RoMed Klinik Bad Aibling, Bad Aibling, Germany

**Keywords:** EndoCATS, Endoscopic thyroid surgery, Remote approach thyroid surgery, Retroauricular approach, Transaxillary approach, ABBA

## Abstract

**Background:**

Thyroid surgery is often performed, especially in young female patients. As patient satisfaction become more and more important, different extra-cervical “remote” approaches have evolved to avoid visible scars in the neck for better cosmetic outcome. The most common remote approaches are the transaxillary and retroauricular. Aim of this work is to compare Endoscopic Cephalic Access Thyroid Surgery (EndoCATS) and axillo-bilateral-breast approach (ABBA) to standard open procedures regarding perioperative outcome and in addition to control cohorts regarding quality of life (QoL) and patient satisfaction.

**Methods:**

In a single center, 59 EndoCATS und 52 ABBA procedures were included out of a 2 years period and compared to 225 open procedures using propensity-score matching. For the endoscopic procedures, cosmetic outcome, patient satisfaction and QoL (SF-12 questionnaire) were examined in prospective follow-up. For QoL a German standard cohort and non-surgically patients with thyroid disease were used as controls.

**Result:**

The overall perioperative outcome was similar for all endoscopic compared to open thyroid surgeries. Surgical time was longer for endoscopic procedures. There were no cases of permanent hypoparathyroidism and no significant differences regarding temporary or permanent recurrent laryngeal nerve (RLN) palsies between open and ABBA or EndoCATS procedures (*χ*^2^; *p* = 0.893 and 0.840). For ABBA and EndoCATS, 89.6% and 94.2% of patients were satisfied with the surgical procedure. Regarding QoL, there was an overall significant difference in distribution for physical, but not for mental health between groups (*p* < 0.001 and 0.658). Both endoscopic groups performed slightly worse regarding physical health, but without significant difference between the individual groups in post hoc multiple comparison.

**Conclusion:**

Endoscopic thyroid surgery is safe with comparable perioperative outcome in experienced high-volume centers. Patient satisfaction and cosmetic results are excellent; QoL is impaired in surgical patients, as they perform slightly worse compared to German standard cohort and non-surgical patients.

Over the past decades, thyroid surgery developed to one of the safest procedures in surgery with low morbidity and mortality. As thyroid surgery is performed in a high number of young and female patients, recently different extra-cervical so called “remote” approaches have evolved. Using these techniques, a visible scar in the neck is avoided for better cosmetic outcome (cosmetics), as quality of life and patient satisfaction become more and more important outcome variables in surgery [1–9]. The most common remote approaches are the transaxillary and retroauricular approach (RA) [4–6, 8, 10–24], both can be used for endoscopic and robotic procedures. All remote-access techniques have unique benefits but also some disadvantages and therefore need a careful patient selection for morphologic and disease specific factors [1–3, 11, 22, 25–27].

The RA was first described by our group in 2008 as the EndoCATS approach and has even spread worldwide [5, 20, 21, 26]. It was also modified to a robotic “face-lift” approach [8, 24]. Its advantage lies in the smaller dissection area due to the short distance between retroauricular incision and the thyroid; specimen up to 60 mL volume can be retrieved through this access, but only unilateral thyroidectomy procedures can be performed [5, 6]. The axillo-bilateral-breast approach (ABBA) was first described in 2003 by Shimazu et al. and can be used for bilateral procedures. The view on the thyroid gland and the important landmarks are comparable to open surgery because of the ventral access after dividing the strap muscles in the midline and an excellent exposure with use of CO_2_—insufflation [13]. Large specimen can be retrieved through the axillar access [4, 8, 12, 13, 15, 17]. Since its development, ABBA was also introduced in Germany by Strik et al, who published their first experience 13 years ago [19]. More recent, experience with these techniques and robotic variants like bilateral-axillary-breast approach (BABA) and the RA steadily improved and they can even be used in oncologic thyroid surgery with central lymphadenectomy in low-risk and localized differentiated thyroid cancer [15, 28, 29]. Complication rates are similar to open thyroid surgery with a comparable oncologic outcome and better cosmetic satisfaction due to the extra-cervical scar [2, 3, 6, 10, 11, 15–17, 21, 25, 27, 29]. In total, endoscopic thyroid surgery and its robotic variants are technically advanced surgical procedures, which should be performed by experienced hands not only because of the prolonged learning curve in comparison to open thyroid surgery [3]. After developing the EndoCATS in our surgical department [5], we introduced ABBA as well for bilateral thyroid pathologies and complete thyroidectomies in 2009. Due to relevant incidence of incidentally found papillary thyroid carcinoma with bilateral or multifocal tumors [30, 31] as well as other bilateral pathologies of the thyroid gland, a scarless endoscopic technique not only for unilateral but also bilateral thyroidectomies is needed in a specialized surgical department.

Our aim in this work was to compare both endoscopic techniques to standard open procedures regarding perioperative outcome and in addition to control cohorts regarding quality of life and patient satisfaction.

## Materials and methods

This study was approved by the local institutional review board. We performed a prospective comparison of the two endoscopic approaches. Fifty-nine EndoCATS and 52 ABBA procedures were included from the same two-year time period. A total of 225 open procedures of a corresponding time period are included for comparison regarding the perioperative outcome.

Perioperative data (patient characteristics, extent of resection, time of surgical procedure, perioperative complications, patho-histologic result) were collected. The extent of resection was sub-divided into near-total, sub-total, total thyroidectomy procedures or Dunhill procedure. Patients who underwent endoscopic surgery were invited for prospective follow-up examination and asked to complete questionnaires for quality of life (SF-12 questionnaire), grading of cosmetic result and patient satisfaction. Perioperative complications were subdivided in general complications (bleeding, temporary or permanent RLN palsy, temporary or permanent hypoparathyroidism) and access-related complications (wound infection, local hematoma, pain or numbness in access area assigned to specific nerves if possible, accidental injury of anatomic structures). Hypoparathyroidism was defined as a drop in blood parathyroid hormone levels (PTH) below regular limits (< 10 ng/mL), regardless of the presence of typical hypocalcemic symptoms. A persistence for longer than six months after surgery, of pain, hypoparathyroidism or RLN palsy were defined as permanent.

The SF-12 questionnaire was chosen, as it is a reliable and validated instrument for life quality measurement and feasible due to its limited number of items [32, 33]. The SF-12 scoring results for physical and mental health composite scales were compared to a collective of patients with known thyroid disease but without surgical therapy from our hospital’s outpatient department and a validated, healthy representative German cohort. A questionnaire for patient’s satisfaction and cosmetic outcome was used (Table 1).

**Table 1:** Questionnaire for patient’s satisfaction and cosmetic outcome

1	Were you satisfied with the thyroid surgery?
	Very satisfied
	Satisfied
	Slightly satisfied
	Not satisfied
2	How satisfied are you with the cosmetic result of this endoscopic thyroid surgery?
	Grade 1 (highest)
	Grade 2
	Grade 3
	Grade 4
	Grade 5
	Grade 6 (lowest)
3	Would you choose this endoscopic technique again?
	Yes
	Maybe
	No

### Preoperative patient preparation and patient selection

The patients were informed about the operative techniques (ABBA, EndoCATS, conventional open thyroid surgery) and allocated to groups depending on the thyroid disease, extent of resection, patients’ preference and medical reasons. Indication for surgery and extent of thyroid resection were decided according to national and International guidelines [34, 35]. All patients signed informed consent for surgery and underwent routine preoperative evaluation using ultrasound, thyroid hormone tests and if needed fine needle aspiration for cytology. A laryngoscopy was performed in all patients to evaluate vocal cord function.

For all surgical procedures neuromonitoring (Avalanche, Dr. Langer Medical, Waldkirch, Germany) was used. For open surgery, mono- and bipolar electrocoagulation and Vicryl sutures (Ethicon, Norderstedt, Germany) were used. The open procedures were performed in a conventional surgical technique. For endoscopic procedures, a standard laparoscopic equipment (Karl Storz SE and Co. KG, Tuttlingen, Germany) and vessel sealing devices (Ultracision, Ethicon Endo-Surgery Inc., J&J Medical Devices, Cincinnati, Ohio, USA) and a bipolar clamp were used. EndoCATS was performed in a modification to our previous description [5, 6, 36], since we used an approach above or ventral the sternocleidomastoid muscle then distally passing between the sternal and clavicular head to reach the surgical space of de Quervain to avoid damage to the accessory nerve [36]. ABBA was performed similar to the technique described by Bährlehner et al. [4]. All patients were screened for hoarseness postoperatively and were advised to undergo laryngoscopy after surgery.

For statistical analysis SPSS Statistics 25 (IBM, Armonk, NY, USA) and for graphical illustration Prism 8 (GraphPad Software, CA, USA) was used. Descriptive statistics and calculation of mean values were used to summarize patient characteristics and perioperative data.

Propensity score (PS) matching was performed to minimize confounding bias and generate more reliable results [37] using XLSTAT (Addinsoft, Paris, France). A 1:1 matching without replacement using a greedy algorithm and a caliper distance of 0.1 standard deviations of the logit of the propensity score was performed. The following preexisting confounders were used for matching because of possible impact on surgical outcome: sex, unilateral vs. bilateral procedures and extent of resection (near-total, sub-total, total or Dunhill procedure). Groups were compared with the non-parametric Mann–Whitney U-test or Kruskal–Wallis one-way analysis of variance (ANOVA) with Bonferroni correction for multiple comparisons, as the Shapiro–Wilk test showed that the metric data were not normally distributed. For comparison of SF-12 data, additionally the effect size after Cohen was calculated as the data at least visually approached a normal distribution. For comparison of data between groups concerning nominal or categorical data, we used the *χ*^2^ test and Fisher’s exact test for analysis with small case numbers. Pearson correlation was used to test relations between metric variables. *P* values ≤ 0.05 were considered statistically significant.

## Results

### Baseline characteristics

An overview of all demographic and perioperative data of the conventional open group before and after PS matching compared to endoscopic groups gives Table 2. Patients treated with the endoscopic techniques are significantly younger (KW: *p* < 0.001) compared to those treated with open surgical technique. After propensity score matching, sex is distributed equally between open and endoscopic procedures (*χ*^2^; *p* = 0.757). While at least a few men (8.5%) were operated with EndoCATS, the ABBA procedure was only performed in female patients because of the bilateral breast approach.

**Table 2:** Baseline demographic and perioperative data of all groups: open procedures, open procedures after propensity score matching (PS matched for: sex, type of resection and extent of resection), all endoscopic procedures, ABBA and EndoCATS procedures

	All open procedures	PS-matched open procedures	All endoscopic	ABBA	EndoCATS
Number procedures/patients	*N* = 225/225	*N* = 111/111	*N* = 111/109	*N* = 52 /52	*N* = 59/57
Age* (KW; *p* < 0.001), (PS:KW; *p* < 0.001)	59.0 ± 12.2 (14–84)	56.5 ± 13.3 (14–77)	46.32 ± 11.1 (19–75)	48.4 ± 12.0 (19–73)	44.5 ± 10.0 (20–75)
Sex (*χ*^2^; *p* < 0.001), (PS:*χ*^2^; *p* = 0.757)					
Female	151 (67.1%)	105 (94.6%)	106 (95.5%)	52 (100%)	54 (91.5%)
Male	74 (32.9%)	6 (5.4%)	5 (4.5%)	0	5 (8.5%)
ASA score* (*χ*^2^; *p* < 0.001), (PS:*χ*^2^; *p* = 0.005)					
1	99 (44.0%)	55 (49.5%)	72 (64.9%)	26 (50.0%)	46 (78.0%)
2	99 (44.0%)	41 (36.9%)	36 (32.4%)	23 (44.2%)	13 (22.0%)
3	27 (12.0%)	15 (13.5%)	3 (2.7%)	3 (5.8%)	0
Type of resection* (*χ*^2^; *p* < 0.001) (PS:*χ*^2^; *p* = 0.043)					
Hemithyroidectomy	74 (32.9%)	54 (48.6%)	69 (62.2%)	10 (19.2%)	59 (100%)
Thyroidectomy	151 (67.1%)	57 (51.4%)	42 (37.8%)	42 (80.8%)	0
Extent of resection (*χ*^2^; *p* = 0.157), (PS:*χ*^2^; *p* = 0.203)					
Total	190 (84.4%)	90 (81.1%)	96 (86.5%)	39 (75.0%)	57 (96.6%)
Subtotal	3 (1.3%)	0	0	0	0
Near total	1 (0.4%)	1 (0.9%)	3 (2.7%)	1 (1.9%)	2 (3.4%)
Dunhill procedure	31 (13.8%)	20 (18.0%)	12 (10.8%)	12 (23.1%)	0

*p* values are corrected by Bonferroni correction in case of multiple comparisons

*Significant at *p* < 0.05 (*KW* Kruskal Wallis one-way ANOVA, *χ*^*2*^) between PS matched open and all endoscopic procedures

The distribution of ASA score differed significantly between open and endoscopic groups (*χ*^2^; *p* = 0.005) due to patient selection of young and predominantly healthy female patients for remote-access thyroid surgery. Rates for uni- and bilateral resections are distributed significantly different between groups despite propensity score matching (*χ*^2^; *p* = 0.043), as a large amount of endoscopically performed procedures are hemithyroidectomies (62.2%). The extent of resection is distributed equally between groups (*χ*^2^; *p* = 0.203), but in EndoCATS group the number of total resections is higher compared to ABBA and open procedures (96.6% vs. 75.0% and 81.1%; *χ*^2^: *p* < 0.001 and 0.001).

### Perioperative data

The perioperative data of PS matched open and all endoscopic procedures are summarized in Table 3. The distribution of resected specimen volume is significantly different in hemithyroidectomies (KW; *p* = 0.008), but only between EndoCATS and open procedures the difference is significant in post-hoc comparison (*p* = 0.006; EndoCATS vs. ABBA: *p* = 1.0; ABBA vs. open: *p* = 0.842). Regarding resected specimen volume, there is no significant difference between open and ABBA thyroidectomies (KW; *p* = 0.058). The histologic results are not distributed significantly different between open and ABBA/EndoCATS procedures (*χ*^2^; *p* = 0.866) and endoscopic groups (*χ*^2^; *p* = 0.572); slightly more nodular goiters and less adenomas have been diagnosed in ABBA group.

**Table 3:** Baseline data of PS-matched open versus ABBA and EndoCATS cohort and perioperative outcome

	PS-matched open procedures	All endoscopic (ABBA + EndoCATS)	ABBA	EndoCATS
Number of procedures	*N* = 111	*N* = 111/109	*N* = 52	*N* = 59
Resected volume				
Total^a^ (KW; *p* < 0.001)	49.8 ± 41.0 (3 –180)	29.1 ± 21.2 (4–102)	40.8 ± 23.2 (5–102)	18.8 ± 12.3 (4–63)
Hemithyroidectomy^a^ (KW; *p* = 0.008)	38.7 ± 40.2 (3–180)	20.2 ± 15.9 (4–102)	27.8 ± 28.2 (5–102)	18.8 ± 12.3 (4–63)
Thyroidectomy (KW; *p *= 0.058)	61.5 ± 38.8 (12–164)	44.3 ± 20.8 (13–94)	44.3 ± 20.8 (13–94)	
Histologic result (*χ*^2^; *p* = 0.866)				
Nodular goiter	82 (73.9%)	80 (72.1%)	40 (76.9%)	40 (67.8%)
Follicular adenoma	17 (15.3%)	18 (16.2%)	6 (11.5%)	12 (20.3%)
Thyreoiditis	5 (4.5%)	7 (6.3%)	3 (5.8%)	4 (6.8%)
Grave’s disease	3 (2.7%)	1 (0.9%)	1 (1.9%)	0
Differentiated carcinoma	4 (3.6%)	5 (4.5%)	2 (3.8%)	3 (5.1%)
Surgical time				
Total^a^ (KW: *p* < 0.001)	141.9 ± 67.1 (61–503)	152.4 ± 50.8 (60–375)	175.9 ± 54.0 (94–375)	131.6 ± 37.5 (60–223)
Hemithyroidectomies (KW: *p* = 0.043)	123.1 ± 54.7 (61–272)	134.4 ± 39.2 (60–223)	150.8 ± 46.4 (94–220)	131.6 ± 37.5 (60–223)
Thyroidectomies^*^ (KW: *p* = 0.003)	157.4 ± 70.3 (67–503)	181.8 ± 54.3 (110–375)	181.8 ± 54.3 (110–375)	
Hospital stay^*^ (KW; *p* = 0.001)	2.6 ± 1.0 (1–7)	2.4 ± 0.8 (1–6)	2.7 ± 0.9 (2–6)	2.2 ± 0.6 (1–5)
Access related complications				
Infection (*χ*^2^; *p* = 0.285)	0	1 (0.9%)	1 (1.9%)	0
Temporary numbness (*χ*^2^; *p* = 0.083)		63 (56.8%)	25 (48.1%)	38 (64.4%) (NAM, NTC)
Superficial hematoma^*^ (*χ*^2^; *p* = 0.022)		43 (38.7%)	26 (50%)	17 (28.8%)
General complications				
Surgical revision for hematoma (*χ*^2^; *p* = 0.920)	3 (2.7%)	4 (3.6%)	2 (3.8%)	2 (3.4%)
Cervical hemorrhage (*χ*^2^; *p* = 0.996)	2 (1.8%)	2 (1.8%)	1 (1.9%)	1 (1.7%)
Temp. Hypoparathyroidism (*χ*^2^; *p* = 0.151)	5 (4.5%)	6 (5.4%)	5 (9.6%)	1 (1.7%)
Perm. Hypoparathyroidism (*χ*^2^; n.a.)	0	0	0	0
Temp. RLN palsy (*χ*^2^; *p* = 0.893)	3 (2.7%)	3 (2.7%)	1 (1.9%)	2 (3.4%)
Perm. RLN palsy (*χ*^2^; *p* = 0.840)	1 (0.9%)	2 (1.8%)	1 (1.9%)	1 (1.7%)
Intraoperative complications (*χ*^2^; *p* = 0.741)	5 (4.5%)	6 (5.4%)	4 (7.7%)	2 (3.8%)

*p* values are corrected by Bonferroni correction in case of multiple comparisons

*NAM* Nervus auricularis magnus, *NCT* nervus transversus colli, *temp* temporary, *perm* permanent

*Significant at *p* < 0.05 (KW: Kruskal Wallis one-way ANOVA, *χ*^2^, *n.a* not available for all values = 0)

The surgical times for hemithyroidectomies are shorter in open compared to endoscopic procedures (KW:* p* = 0.043), but without significant difference between open and EndoCATS or ABBA procedures in post-hoc comparison (KW; *p* = 0.139 and *p *= 0.126). The surgical time is significant longer for ABBA compared to open thyroidectomies (KW*: p* = 0.003). For thyroidectomies or hemi-thyroidectomies, there is no correlation of surgical time and volume of the resected specimen (*r *= 0.10 and 0.01). One-way ANOVA showed a significant difference between groups regarding length of hospital stay (KW; *p* = 0.001). In post-hoc multiple comparison hospital stay was significantly shorter in EndoCATS versus ABBA and EndoCATS versus open thyroid surgeries (*p* = 0.002 and 0.003), but not in ABBA vs. open procedures (*p* = 1.0).

### Perioperative complications

The perioperative outcome is summarized in Table 3. There were no conversions from endoscopic to open surgery. Only one wound infection was observed in an axillary wound after an ABBA procedure; the wound had to be opened to heal by secondary intention (1.9%). Temporary numbness in 48.1% and paresthesia in 64% along the access route to the thyroid space were observed in both ABBA and EndoCATS procedures without a significant difference between groups (*χ*^2^; *p* = 0.083). Local access related but clinical not relevant hematoma was found significantly more often in ABBA 50.0% than in EndoCATS 28.8% procedures (*χ*^2^; *p* = 0.022).

There was one case of acute hemorrhage in each endoscopic group, which needed surgical revision on the day of surgery. In the EndoCATS case (1.7%) diffuse bleeding could be managed endoscopically. The ABBA patient developed a swelling and “globus sensation”. The hematoma could be released by a small incision at bedside. The rates for bleeding complications showed no difference between open and ABBA/EndoCATS procedures (*χ*^2^; *p* = 0.708). In open procedures there were *n* = 3 cases of bleeding complications with need for revision (2.7%), only two were urgent, whereas in one case revision surgery was carried out on 6th POD without signs of potentially fatal hemorrhage. In ABBA procedures there was one surgical removal of a hematoma along the access site (1.9%) and in the EndoCATS group we performed one surgical revision of the access (1.7%) along the sternocleidomastoid muscle to release local symptoms on POD 2.

No case of permanent hypoparathyroidism occurred. The rates of temporary hypoparathyroidism showed no significant difference between the open and endoscopic groups (*χ*^2^; *p* = 0.151), although the rates were higher in ABBA and open procedures (9.6% vs. 4.5% vs. 1.7%). Temporary hypoparathyroidism occurred significantly more often in thyroidectomies compared to hemithyroidectomies irrespective of open or endoscopic access (*χ*^2^; *p* = 0.011).

There are no significant differences regarding temporary/permanent RLN palsies between open (2.7%/0.9%), ABBA (1.9%/1.9%) /EndoCATS (3.4%/1.7%) procedures (*χ*^2^; *p* = 0.893/0.840) and when comparing the two endoscopic groups (*χ*^2^; *p* = 0.634/0.928). Neither the extent of resection nor the type of surgical procedure (thyroidectomies vs. hemithyroidectomies) showed a significant impact on the rates of RLN palsies (*χ*^2^; *p* = 0.071 and 0.610) or bleeding complications (*χ*^2^; *p *= 0.488/0.834).

The rates of intraoperative complications like loss of signal using neuromonitoring or major bleeding was without difference between the open and EndoCATS/ABBA procedures (*χ*^2^; *p* = 741), nor between the two endoscopic groups (*χ*^2^; *p* = 0.495).

### Follow-up and patient-reported outcome measures

Follow-up rate for ABBA and EndoCATS procedures was 92.3% and 88.1% respectively (*χ*^2^; *p* = 0.321). Duration of follow-up was longer for ABBA (23.7 ± 12.2 month) than for EndoCATS procedures (15.0 ± 7.2 month; MW: *p* < 0.001) (Table 4). Only 60.4% of ABBA and 50% of EndoCATS patients had a laryngoscopy performed postoperatively (*χ*^2^; *p *= 0.011). All patients with perioperative major complications (bleeding complications, temporary hypoparathyroidism, intra operative loss of signal or RLN palsy) took part in follow-up. Patients lost to follow-up all belonged to the group with an uneventful intra- and postoperative course. All patients with loss of signal during intra operative nerve monitoring had at least one postoperative laryngoscopy, therefore, only patients with an intact RLN and vagus nerve signal in neuromonitoring were lost to follow-up.

**Table 4:** Follow-up and long-term outcome for endoscopic groups

	ABBA	EndoCATS
Number procedures/patients	*N* = 52	*N* = 59/57
Follow-up rate(χ^2^; *p* = 0.321)	48 (92.3%)	52 (88.1%)
Follow-up duration (month)^a^ (MW; *p* < 0.001)	23.7 ± 12.2 (6–28)	15.0 ± 7.2 (8–42)
Postoperative laryngoscopy^a^ (*χ*^2^; *p* = 0.011)	29 (60.4%)	26 (50%)
Patient satisfaction (*χ*^2^; *p* = 0.191)		
Very satisfied	34 (71%)	45 (86%)
Satisfied	9 (19%)	4 (8%)
Slightly satisfied	5 (10%)	2 (4%)
Not satisfied	0	1 (2%)
Rating of cosmetic result (*χ*^2^; *p* = 0.506)		
1	37 (78%)	43 (84%)
2	8 (18%)	5 (10%)
3	1 (2%)	0
4	1 (2%)	2 (4%)
5	0	0
6	0	1 (2%)
Would patients choose this surgical technique again (*χ*^2^; *p* = 0.426)		
Yes	39 (82%)	46 (88%)
Maybe	5 (10%)	2 (4%)
No	4 (8%)	4 (8%)

^a^Significant at *p* < 0.05 (MW Mann Whitney U test, *χ*^2^)

89.6% and 94.2% of patients, respectively, were satisfied with the surgical procedure in the ABBA and the EndoCATS groups without significant difference in distribution between endoscopic groups (*χ*^2^; *p* = 0.191). The cosmetic result was rated with best two grades in 95.7% and 94.1% of patients in ABBA and EndoCATS procedures; there was no significant difference in distribution between groups (*χ*^2^; *p* = 0.506). 92.7% of ABBA and 92.3% of EndoCATS patients would consider undergoing endoscopic surgery by the same technique again (*χ*^2^;* p* = 0.426), whereas only 8.3% of ABBA and 7.7% of EndoCATS patients would refuse this (Table 4).

Regarding QoL by SF-12 questionnaire, there was an overall difference for physical but not for mental health composite scales between the groups (KW; *p* < 0.001 and 0.658). In post-hoc multiple comparison there was no significant difference between the non-surgical control group vs. EndoCATS (*p* = 0.528) and ABBA (*p* = 0.398), but between EndoCATS and ABBA compared to the German reference cohort (*p* = 0.002 and 0.001). As shown in Table 5 and Fig. 1, the endoscopic groups perform slightly worse regarding physical health compared to the non-surgical patients and German reference cohort; this effect was only small according to the effect size for ABBA (*d* = − 0.359) and EndoCATS (*d* = − 0.323).

**Table 5:**
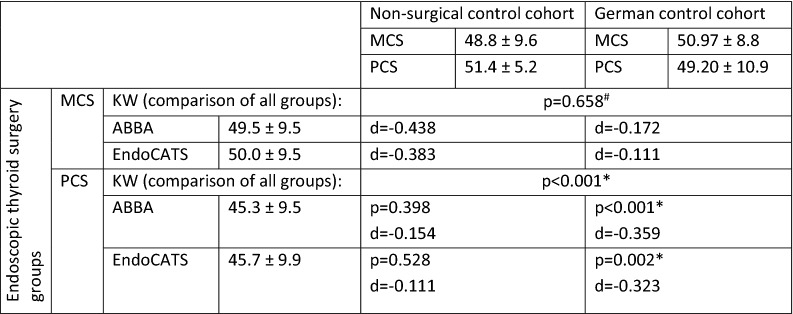
Quality of life according to SF-12 questionnaire and comparison between MCS and PCS groups with Kruskal Wallis one-way ANOVA with post-hoc multiple comparisons

Mean values ± standard deviation

*MCS* mental health composite scale, *PSC* physical health composite scale

*Significant at *p* < 0.05 (KW: Kruskal Wallis one-way ANOVA; d = effect size; all *p*-values are corrected by Bonferroni correction for multiple comparisons)

#No p values available for comparison between separate groups as KW shows no overall significant difference for MCS and therefore no post-hoc multiple comparison was performed

**Fig. 1:**
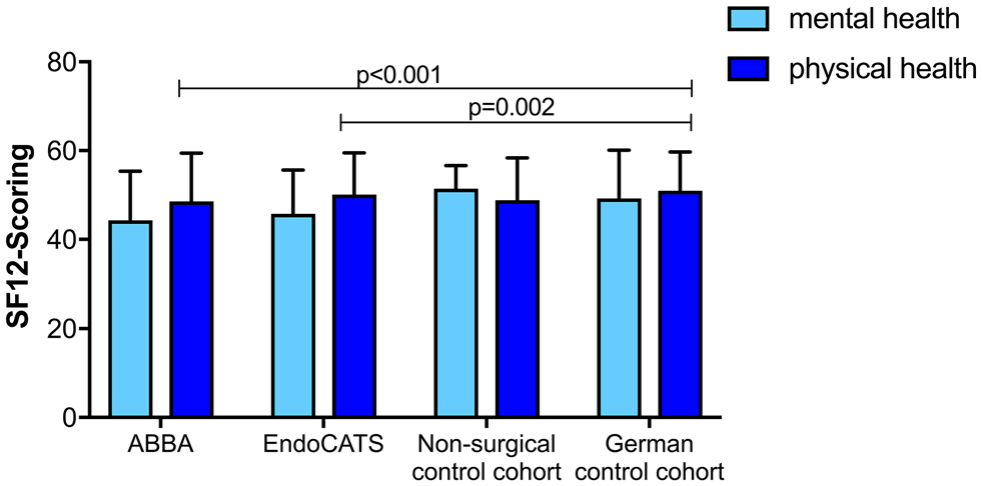
SF-12 scores for EndoCATS, ABBA, non-surgical control and German reference cohorts: comparison by Kruskal–Wallis one-way ANOVA: mental health composite scale: *p* = 0.658, physical health composite scale: *p* < 0.001 (only significant *p*-values are shown for post-hoc multiple comparison between groups)

## Discussion

Here, we present for the first-time clinical results directly comparing two remote approaches in thyroid surgery regarding perioperative outcome and quality of life. Remote access endoscopic thyroid surgery has recently been shown to be safe and efficient not only in treatment of benign disease, but also for differentiated thyroid cancer [3, 7, 9, 20, 38].

We were able to show a decent peri- and postoperative outcome following the endoscopic procedures comparable to conventional open thyroid resections. The complication rates were very similar for all three groups, with no cases of permanent hypoparathyroidsm and only one case of permanent RLN palsy in each of the ABBA and EndoCATS groups. Acute hemorrhage occurred in only one case of each group, as well. Only in ABBA group there was one case of wound infection, which needed surgical revision. In both endoscopic remote access techniques, there was a rather a high rate of specific access related complications like numbness or superficial hematoma with skin discolorations, which were, however, only temporary in nature. Such local complications have also been described in conventional open thyroidectomy procedures. Here they are, however, usually not of special interest since these are well established standard of care techniques [10, 12, 25, 38, 39]. We used endoscopic techniques in a highly selected patient collective, most patients underwent surgery for suspicious or symptomatic thyroid nodules or inflammatory thyroid disease.

Other groups report less favorable results using a RA, especially regarding the rates of RLN palsies (up to 4.4%), bleeding complications (up to 4.8%) with longer in hospital stays of about 4.4 days [12, 16] and similar surgical time for hemithyroidectomies of about 129 min by mean [12]. These groups use a much larger skin incision creating a skin flap predominantly in robotic—but also in endoscopic RA thyroid surgery techniques [8, 11, 24, 26]. Lira et al. report their results in robotic RA thyroid surgery, making up only a small fraction of 1.7% of all thyroidectomy procedures, a high rate of female patients (90%) with a mean age of 35 years and rates of 6.2% for temporary RLN palsy and 6.2% of temporary hypocalcemia [20]. Lee et al. compared the RA approach to the transaxillary one in hemithyroidectomies only with a similar sample size [12]. In our department, we use the RA usually for unilateral endoscopic cases. This technique has its obvious limitation, mostly due to the single port approach and limited surgical space as the midline is not opened like in ABBA, open or transoral techniques. However, with the advantage of a smaller dissection area and an excellent view on the RLN and the parathyroids. We use the transaxillary ABBA approach mostly for bilateral resections and in some unilateral cases with large specimen sizes or in rare situations, if patients prefer ABBA to EndoCATS approach. In our data the number of Dunhill, total, sub- and near-total resections were distributed equally over the groups, only the size of the resected specimen was smaller especially in EndoCATS procedures but also in the ABBA group compared to conventional open hemi- and thyroidectomies; only the difference for hemithyroidectomies was significant.

For ABBA and other transaxillary approaches comparable complication rates have been reported in literature with rates of RLN palsy between 0 and 3.4%, bleeding complications around 3.5% and temporary hypocalcemia in up to 23% of cases [15, 16, 18]. Independent of surgical technique, rates of temporary hypoparathyroidism were expectedly higher in thyroidectomies compared to hemithyroidectomies, but especially in ABBA technique the rate was quite high (9.6%).

EndoCATS and ABBA are very different procedures that can only be compared to some extent. In our opinion, they are complementary, which enables us to offer our patients a tailored solution regarding personal preference, surgical technique and an approach depending on underlaying disease and extent of indicated surgical procedure. Despite the fact that the perioperative outcome of endoscopic “remote access” thyroid surgery can be considered favorable regarding the common complications, all experts advise to pay attention to a meticulous patient selection, to ensure an adequate expertise in high volume centers and to respect the indications for thyroid surgery as given by the national and international guidelines [2, 3, 34, 35]. Taken together these recommendations and our experience, we suggest remote-access procedures for young patients without relevant comorbidities who want to avoid a visible scar in the neck. The thyroid pathologies should be well-circumscribed like suspicious nodules including early stage differentiated thyroid cancers without any sign of extrathyroidal extension or lymph node involvement.

In both of our endoscopic groups 3.8% (ABBA) and 5.1% (EndoCATS) of the cases, respectively, were histologically diagnosed with differentiated thyroid carcinoma. Four of the five cases were sufficiently treated with the endoscopic procedure alone. Only one case with a T3 papillary thyroid carcinoma needed open revision with central lymph node dissection, which wouldn’t have been possible using the unilateral RA. Other groups mostly in the Asian countries have been using endoscopic and robotic techniques in treatment of differentiated thyroid cancer for years with excellent outcomes. They do not only perform thyroidectomies but also central and lateral lymph node dissections with similar outcomes compared to conventional open procedures regarding lymph node retrieval and long-term patient survival [25, 28, 38, 40, 41]. Altogether, the endoscopic techniques turned out to be safe and effective not only in benign thyroid disorders, but also for limited thyroid malignancies. Nonetheless, they are not recommended for treatment of thyroid cancer by German or International guidelines [34, 35]. The *American Thyroid Association* published a statement on remote access surgery in 2016 considering extrathyroidal spread or lymph node involvement as clear contraindications for remote access thyroid surgery. On the other hand, they stated a favorable outcome not only for papillary microcarcinoma but also for other differentiated thyroid cancers and report at least comparable number of retrieved lymph nodes in robotic thyroid cancer surgery [2].

A clear advantage of the remote access techniques compared to conventional open thyroid surgery is the cosmetic result without a visible scar in the neck. Therefore, patients undergoing endoscopic thyroid surgery were significantly younger, healthier and the number of female patients was significantly higher compared to standard open procedures, the latter before propensity score matching. More than 90% of our patients in both endoscopic groups were satisfied with the cosmetic result. Only 2% of patients were not satisfied in the EndoCATS group. The cosmetic result was graded with two best grades (1 or 2) in more than 94% of patients in both endoscopic groups. Only 8% of patients in both endoscopic groups would not undergo the same surgical procedure again. Regarding the rating of cosmetic results, no comparable data are available in literature for endoscopic or remote access thyroid surgery. Patient satisfaction was quite high in our endoscopic groups. Satisfaction rates between 76 and 99% are also reported by Chung for RA and Miccoli for MIVAT procedures [7, 39]. Remote access thyroid surgery is mostly asked for by young female patients [3, 8, 10, 25, 41, 42] making up 95% of all patients in our study. The patients asking for endoscopic and in general remote access thyroid surgery without visible scars in the neck are a highly selective collective of patients with high expectations and a clear, if certainly idiosyncratic, idea of physical integrity.

In contrast to other available studies on perioperative outcome and cosmetic or general patient satisfaction with endoscopic or remote access thyroid surgery, we additionally used the SF-12 questionnaire to objectify our patient’s quality of life. For endoscopic or robotic thyroid surgery there are yet no comparable data available. Our patients showed a similar QoL between the two endoscopic groups and our controls in follow-up at least regarding mental health. For physical health composite scales, we could detect a statistical difference between endoscopic groups and German control cohort with significance in the post-hoc multiple comparison between single groups, but only small effect size, indicating a slightly worse outcome. We lack data on preoperative quality of life of our patients and for the open thyroid procedures, so that a more in-depth comparison of the groups is not possible. Some recent published studies address the question of QoL after open thyroid surgery. Van Velsen et al. showed a significant decrease of QoL after surgery which took up to 3 years to normalize to the baseline scoring in a cohort of 185 patients with differentiated thyroid cancer [43]. In women with benign euthyroid goiter, Promberger et al. did not find an overall improvement in health related QoL after undergoing thyroid surgery [44]. In a recently published randomized controlled trial, QoL significantly increased after surgery in a cohort with euthyreotic Hashimoto’s disease compared to a cohort only receiving hormone substitution alone [45]. Altogether, QoL data in thyroid surgery are sparse to find in literature and in the available data most patients perform worse in different aspects of QoL with thyroid disease and even after thyroid surgery for years in benign or malign disease [43, 44, 46–48]. Therefore, the decrease especially in physical health composite scale, may not be due to the surgery itself. Based on the sparse data on QoL presently available [43–46, 48] it might be related to a problem in mostly young and female patients, who are impaired by thyroid disease in general.

The SF-12 QoL as well as the more detailed SF-36questionnaires are widely used as validated and reliable tools, which can help to objectify patient’s QoL. They seem, however, unspecific regarding endocrinologic or especially thyroid disorders, since QoL depends not only on clinical but also on socio-demographic factors [33]. The data published so far lack consistency, as they all use different tools for evaluation of QoL or patients’ satisfaction. Nonetheless, these patient-reported outcomes become more and more important, especially for the evaluation of newly developed surgical techniques. Recently, a specialized tool named “*thyroid-related quality of life—ThyPRO*” for thyroid disease-related QoL was published [49]; a widespread use of the same established QoL tool in thyroid disease and surgery would be helpful for reliable comparisons of available, not only surgical, treatment options.

There are several limitations in this work. One is that we reported the outcome of only a small number of patients in a highly selected collective from a single center. However, other groups also report only a small number of cases with a low degree of evidence [8, 10, 12, 18, 20, 21, 25, 39–42]. Furthermore, there is a bias due to patient selection for either one of the endoscopic or open procedures, as the EndoCATS approach can only be used for unilateral resections, whereas the ABBA technique is suitable for uni- and bilateral resections but only in female patients. Therefore, propensity score matching was used between open and endoscopic procedures to minimize confounding bias and increase reliability of the here presented results [37]. Nonetheless, the endoscopic surgical techniques were used in a highly selected much younger, healthy cohort with a high number of female patients with selected thyroid pathologies and small resection volumes. Therefore, after propensity score matching some inhomogeneity remained between the open and endoscopic groups especially regarding type of resection, as with the endoscopic techniques in total 62% of procedures have been hemithyroidectomies compared to 33% in the conventional open baseline cohort. The prospective follow-up was only available for about 90% of our endoscopically treated patients, so we cannot exclude some selection bias regarding the patient reported data dealing with patient satisfaction, rating of the cosmetic result and QoL. Furthermore, only about 50% of patients performed the recommended postoperative laryngoscopy, but at least all patients with RLN palsy or intraoperative loss-of-signal in neuromonitoring had at least one postoperative laryngoscopy performed. As we documented patient satisfaction, rating of the cosmetic result and QoL data for the endoscopic cohort prospectively after surgical intervention only, no comparable data for conventional open surgical procedures are available for a more comprehensive comparison.

Despite the fact, that the endoscopic techniques offer an excellent view on the surgical field due to video-endoscopy and the associated enlargement effect, these procedures are known to have a prolonged learning curve and for patient’s safety need to be performed by a specialized surgeon in high-volume centers [3, 11, 16]. On the other hand, only specimen of limited volume can be retrieved due to the remote approach and limited skin incisions; therefore, not all patients are suitable for these remote approach techniques. Surgical times are longer in endoscopic compared to open thyroid surgery, but time seems to decrease after completing the learning curve [16]. Neither in Germany nor internationally, the remote access procedures have not yet been included in the guidelines for the treatment of at least differentiated thyroid cancer. In Asian countries, however, endoscopic techniques are already well established not only for the radical resection of cancer but also for central as well as selective lateral lymph node dissection in thyroid cancer patients [11, 25, 28, 29, 38, 40, 41, 50]. In Europe there have been some first successful experiences with the transaxillary robotic approach in selected patients with differentiated thyroid cancer for thyroid resection and central lymph node dissection [51].

## Conclusion

With regard to the recently published data on remote access thyroid surgery, a tailored approach with a feasible and save procedure associated with good cosmetic results for at least benign thyroid disease in a mostly young and female population is possible using different techniques of endoscopic thyroid surgery in a single high-volume center. Remote access endoscopic thyroid surgery like EndoCATS and ABBA procedures can be safely performed with an excellent cosmetic outcome and highly satisfied patients. The QoL in these patients is impaired, but presumably independent of surgery or surgical technique [43, 44, 46, 47].
